# Treatment of Typhoid Fever with Tar Water

**Published:** 1856-04

**Authors:** 

**Affiliations:** Angoulême.


### ARTICLE XVIII.

*Treatment of Typhoid Fever with Tar Water.* By Dr. Chapelle, of Angoulême. (Translated for the Charleston Medical Journal and Review.)

Having observed the favorable effect of tar in a certain case of typhoid fever, Dr. C. was induced to pay particular attention to this remedy, in a series of cases occurring during the typhoid epidemic of 1854, 1855. His conclusion is, that liquid tar, if not an absolute specific, is yet incontestibly the most efficacious agent yet discovered for the treatment of the above mentioned disease. The tar should be administered internally as a drink, and in the form of an injection.

The drink is prepared in the following manner: About ℥ ij of liquid tar are put into a vessel, containing nearly a quart of hot water; after it has stood a few hours, the patient commences to drink it, filling up with ordinary water after each draught, so that the same dose of tar will last during the whole treatment. The injection is prepared by rubbing up the yellow of one or two eggs with a table-spoonful of liquid tar, and diluting with a little more than a pint of warm water; this serves for two injections.

The patient should drink as much of the draught as he can; as to the injection, that should be insisted on in proportion as the drink disgusts, for the intestines should be always kept supplied with a certain quantity. Sometimes six, eight, and even ten enemata should be administered in twenty-four hours. Should the patient be taken with diarrhea, these injections check it promptly.

This treatment, if continued for two or three days, generally triumphs over the typhoid state; typhoid fever of ordinary intensity, called usually mucous fever, needs double that time; but typhoid fever, properly so-called, of whatever form, is vanquished in its essential phenomena in eight to ten days. Each day the skin loses its dryness and heat, the tongue becomes clearer, the abdomen presents less tension and susceptibility, the sleep is calmer, the fœcal matter acquires a more normal odor, and the digestive functions recover strength. When there exists only a simple typhoid state, the tar draught alone is commonly sufficient; but when the general perturbation augments, the febrile reaction increases, and the functional disorder is excessive, a much stronger dose of the tar is required, and the injections are then indispensable. In all cases where the breast or the head has been affected with violent perturbation, the disappearance of the ordinary typhoid phenomena does not immediately produce a cessation of these complications. These functional disorders either disappear gradually of themselves, or need the application of treatment appropriate to the morbid state.—*Rev. Medico. Chirurgicale.*

